# Supplementary material for: Response to Stimulations Inducing Circadian Rhythm in Human Induced Pluripotent Stem Cells
Source: Cells. 2020 Mar 4;9(3):620. doi: 10.3390/cells9030620 (PMC7140533; doi:10.3390/cells9030620)
Supplement: Supplementary file 1 [file cells-09-00620-s001.zip › Supplementary files/Supplementary Figure S1.pdf]

iPS

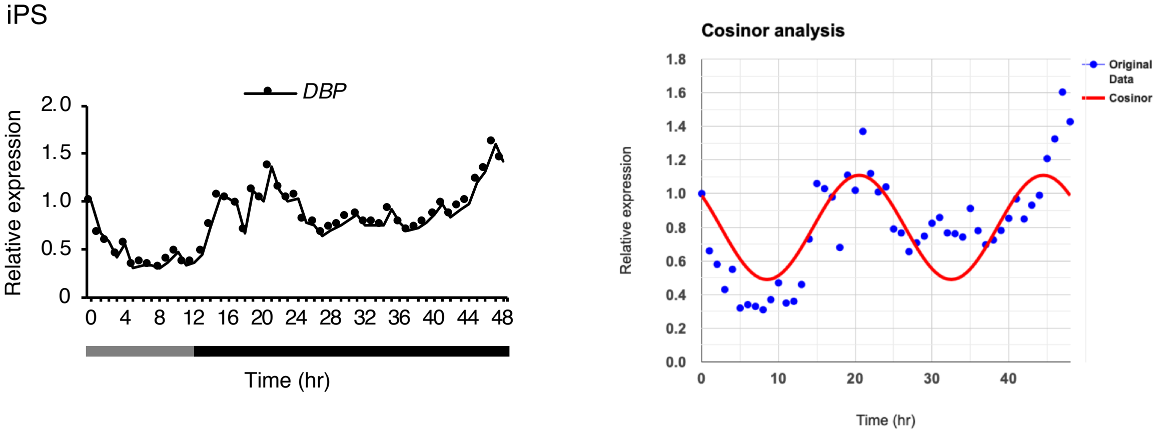

Supplementary Figure S1. The response of *DBP* to the simulated rhythm of body temperature. Left, the expression levels of *DBP* under the temperature rhythm was measured in hiPSCs. 0 h refers the start of day 5 of the temperature rhythm. The temperature was set at 33 ^{o}C from 0 to 12 h (gray bar) and then at 37 ^{o}C from 12 to 48 h (black bar). Data are presented as the means ± SEM relative to 0 h. n = 2. *P* value for the rhythmicity of *DBP* expression was determined by cosinor analysis and it was $p < 0.01$. Right, red line indicates the significant cosinor function fit analyzed from left data.
